# Effectiveness of exhaled nitric oxide for the prediction of non-invasive left atrial pressure in older people: a cross-sectional cohort study

**DOI:** 10.3399/BJGPO.2022.0105

**Published:** 2022-12-14

**Authors:** Samuel Thomas Jones, Monica Londahl, Anthony Prothero, FD Richard Hobbs, Ian Pavord, Saul G Myerson, Bernard D Prendergast, Sean Coffey

**Affiliations:** 1 Department of Medicine, University of Otago, Dunedin, New Zealand; 2 Department of Cardiology, Oxford University Hospitals NHS Trust, Oxford, UK; 3 Nuffield Department of Primary Care Health Sciences, University of Oxford, Oxford, UK; 4 Department of Cardiovascular Medicine, University of Oxford, Oxford, UK; 5 St Thomas’ Hospital, London, UK

**Keywords:** cardiology, heart failure, primary health care, general practice, family practice

## Abstract

**Background:**

During left-sided heart failure (HF), left atrial and pulmonary venous pressure increase, which may lead to pulmonary congestion. Previous cohort studies, examining participants with symptomatic HF or rheumatic heart disease, suggest a relationship between increased left atrial pressure (LAP) and fractional exhaled nitric oxide (FeNO).

**Aim:**

To examine the strength of association between FeNO and echocardiographic assessment of LAP by the E/e’ ratio, to determine if FeNO could be used to identify those with elevated LAP.

**Design & setting:**

This cross-sectional cohort study examined a subset of the OxVALVE cohort aged ≥65 years. Data collection was undertaken in primary care practices in central England.

**Method:**

Each participant underwent a focused cardiovascular history and clinical examination. Standard transthoracic echocardiographic (TTE) assessment was performed on all participants, with the E/e’ ratio calculated to obtain a validated surrogate of LAP. FeNO was measured in 227 participants.

**Results:**

FeNO was higher in males compared with females and no different in participants with asthma, chronic obstructive pulmonary disease (COPD), or those using inhaled steroids. Participants with a high E/e’ (>14) were older, with a higher proportion of females than males. There was no relationship between E/e’ and FeNO, either when measured as a continuous variable or in the group with high E/e’.

**Conclusion:**

FeNO was not found to be an accurate predictor of elevated LAP in a primary care setting.

## How this fits in

Previous cohort studies examining patients with symptomatic HF or rheumatic heart disease suggest a relationship between increased LAP and FeNO. High LAP can frequently be reduced with diuretic therapy, so a simple method of predicting LAP could help guide management in outpatient settings. As FeNO can be measured with a hand-held analyser, a diagnostic test for HF in primary care could be developed if such a relationship exists. The study found that E/e’ ratio measured on echocardiography was not associated with FeNO in a population-based setting; FeNO does not predict LAP in primary care.

## Introduction

HF is a major cause of morbidity and mortality in the UK and worldwide.^1,2^ Approximately 50% of patients with HF present with a left ventricle that essentially contracts normally, termed HF with preserved left ventricular ejection fraction (HFpEF).^3^ Early diagnosis of HF is difficult despite its high prevalence, particularly in older people, with symptoms of HF, such as fatigue and shortness of breath, being frequently seen because of other diagnoses.^4^ Assessment of LAP in these patients is traditionally carried out by echocardiography or invasive cardiac catheterisation. Formal echocardiography requires specialist training and expertise, and is therefore not widely used in the primary healthcare setting. Furthermore, even when left ventricular ejection fraction (LVEF) is measured using point-of-care echocardiography, LAP assessment requires Doppler measurement that is often unavailable on point-of-care devices. A simple cost-effective test for elevated LAP requiring minimal expertise could therefore lead to improved HF diagnosis and identify those patients most likely to benefit from medical therapy.

FeNO may be a suitable diagnostic marker for elevated LAP. In left-sided HF (whether owing to reduced ejection fraction [HFrEF]) or HFpEF), the left ventricle is unable to adequately pump blood to the systemic circulation, leading to elevated LAP^5^ and a subsequent increase in pulmonary venous pressure, resulting in pulmonary congestion and a possible increase in FeNO.^6^ Nitric oxide (NO) is a potent vasodilator^7^ whose production may be *'a compensatory response to increased flow in the pulmonary venous circulation'* in left-sided HF.^6^ Measurement of FeNO is frequently used for the assessment of inflammatory airways disease and portable hand-held FeNO analysers are now inexpensive and reliable,^8^ allowing testing in the primary healthcare setting. If FeNO is a reliable predictor of elevated LAP, it could be used as a simple adjunct to current HF assessment algorithms. Therefore, this study sought to determine whether FeNO can identify subjects with an elevated LAP (E/e' ratio).

## Method

### Participants

This cross-sectional cohort study examined a subset of the original OxVALVE cohort recruited in 2014 and 2015.^9^ The OxVALVE participants are an extensively characterised population-based cohort aged ≥65 years who were invited to attend screening examination and echocardiography at their local primary care (general) practice in central England, UK. Those with a previous diagnosis of valvular heart disease, a terminal illness, excessive frailty, or inability or unwillingness to provide consent were excluded.^9^ This subset of participants was recruited consecutively (once the appropriate testing equipment was available) to examine this specific study question, with no additional inclusion or exclusion criteria applied. Four separate general practices took part in this substudy. All participants provided written consent.

### Clinical and echocardiographic assessment

Demographic and clinical information was collected from each participant, combined with a focused cardiovascular history and clinical examination. The cardiovascular history consisted of past medical conditions (such as asthma, COPD, diabetes, and hypertension), past surgical procedures (such as coronary artery bypass grafting [CABG] and percutaneous coronary intervention [PCI]), smoking history (never smoked, ex-smoker [quit >1 year ago], or current smoker [smoked in past 12 months]), current medications (including inhaled steroids), and the presence of HF symptoms (New York Heart Association classification [NYHA]). Clinical examination included blood pressure, heart rate, height, and weight.

Standard TTE was performed on all participants by a British Society of Echocardiography (BSE)-accredited sonographer or physician using a Vivid-Q portable echocardiographic machine. The reference standard for FeNO was the E/e’ ratio, a validated measure of LAP,^5^ calculated by dividing the TTE-derived mitral E velocity (E) by the septal e’ velocity (e’). Mitral E velocity measures mitral valve flow velocity in early diastole, while septal e’ measures tissue velocity at the mitral valve annulus and is an assessment of vertical relaxation of the base of the heart during diastole. Both measurements were performed using standard methods in the apical four-chamber view, with E velocity measured using pulse wave Doppler at a sample volume placed between the mitral leaflet tips, and septal e’ velocity measured using pulse wave tissue Doppler imaging at a sample volume in the basal septum.

A consecutive subset (nested within the main OxVALVE cohort) of 277 participants underwent FeNO measurement in parts per billion (ppb) using a portable electrochemical analyser (NIOX VERO Airway Inflammation Monitor) and variables with a previously observed association with FeNO examined. E/e’ ratio was grouped into normal (<8), intermediate (8–14), and high (>14) strata.^10^

### Statistical analysis

Owing to non-normal distribution, FeNO was compared in participants matched according to clinical factors (sex, asthma, COPD, smoking status, diabetes mellitus, and use of inhaled steroids) previously identified as affecting FeNO levels (see Discussion) using a Mann-Whitney *U*-test (or Kruskal-Wallis test for the three-level smoking variable). FeNO was compared across E/e’ strata using a Kruskal-Wallis test. Categorical variables are expressed as proportions and percentages, and were compared across E/e’ strata using a χ^2^ test. Associations between FeNO and age, and FeNO and E/e’ (as a continuous variable) were assessed using univariate linear regression. No multivariate adjustment was performed since there were so few significant univariate associations between FeNO and demographic, echocardiographic, and HF risk factors. Statistical analysis was performed using the R statistical computing package (version 3.5.1) and a *P*-value <0.05 considered significant.

## Results

### FeNO and clinical characteristics

The OxVALVE subset was predominately White, 45.1% female (Table 1), and had a mean age of 73.5±6.3 years. There were eight (2.9%) current smokers, 20 (7.2%) participants with asthma, and 16 (5.8%) participants with COPD. Only 10 (3.6%) participants were in NYHA class III. Measurement of FeNO was attempted in a total of 277 consecutive participants and successful in 227 (81.9%). There were no significant differences in major demographic variables between those who could and could not successfully complete a FeNO measurement (see Supplementary Table S1).

**Table 1.** Clinical characteristics, heart failure risk factors, and echocardiographic measurements in the OxVALVE subset

Characteristic	Total cohort, *n* (%)^a,b^
Participants	277 (100)
Age, years, median (IQR)	73 (68–78)
Sex	
Male	152 (54.9)
Female	125 (45.1)
Ethnic group	
White	269 (97.1)
Asian	5 (1.8)
Black	2 (0.7)
Mixed	1 (0.4)
Smoking status	
Non-smoker	133 (48.0)
Ex-smoker	136 (49.1)
Current	8 (2.9)
BMI, kg/m^2^, median (IQR)	27.6 (24.5–30.4)
Asthma	20 (7.2)
COPD	16 (5.8)
Inhaled steroids	12 (4.3)
Atrial fibrillation	39 (14.1)
Diabetes	38 (13.7)
Hyperlipidaemia	134 (48.4)
Hypertension	149 (53.8)
Myocardial infarct	12 (4.3)
PCI	11 (4.0)
CABG	5 (1.8)
Chronic kidney disease	35 (12.6)
NYHA class	
I	178 (64.3)
II	89 (32.1)
III	10 (3.6)
Socioeconomic class	
1 (least deprived)	67 (24.2)
2	65 (23.5)
3	85 (30.7)
4	36 (13.0)
5 (most deprived)	21 (7.6)
Missing	3 (1.1)
Ejection fraction, %, median (IQR)	69 (66–73)
E velocity, median (IQR)	0.60 (0.51–0.71)
Septal e’ velocity, median (IQR)	0.05 (0.04–0.07)
E/e’, median (IQR)	10.7 (9.1–13.5)
FeNO, ppb, mean (SD)	24.2 (15.6)

^a^FeNO measurement was unsuccessful in 50 participants — these participants' data are included in all variables outside of FeNO. ^b^Unless otherwise stated. BMI = body mass index. CABG = coronary artery bypass grafting. COPD = chronic obstructive pulmonary disease. FeNO = fractional exhaled nitric oxide. NYHA = New York Heart Association. PCI = percutaneous coronary intervention. ppb = parts per billion.

**Figure 1.**
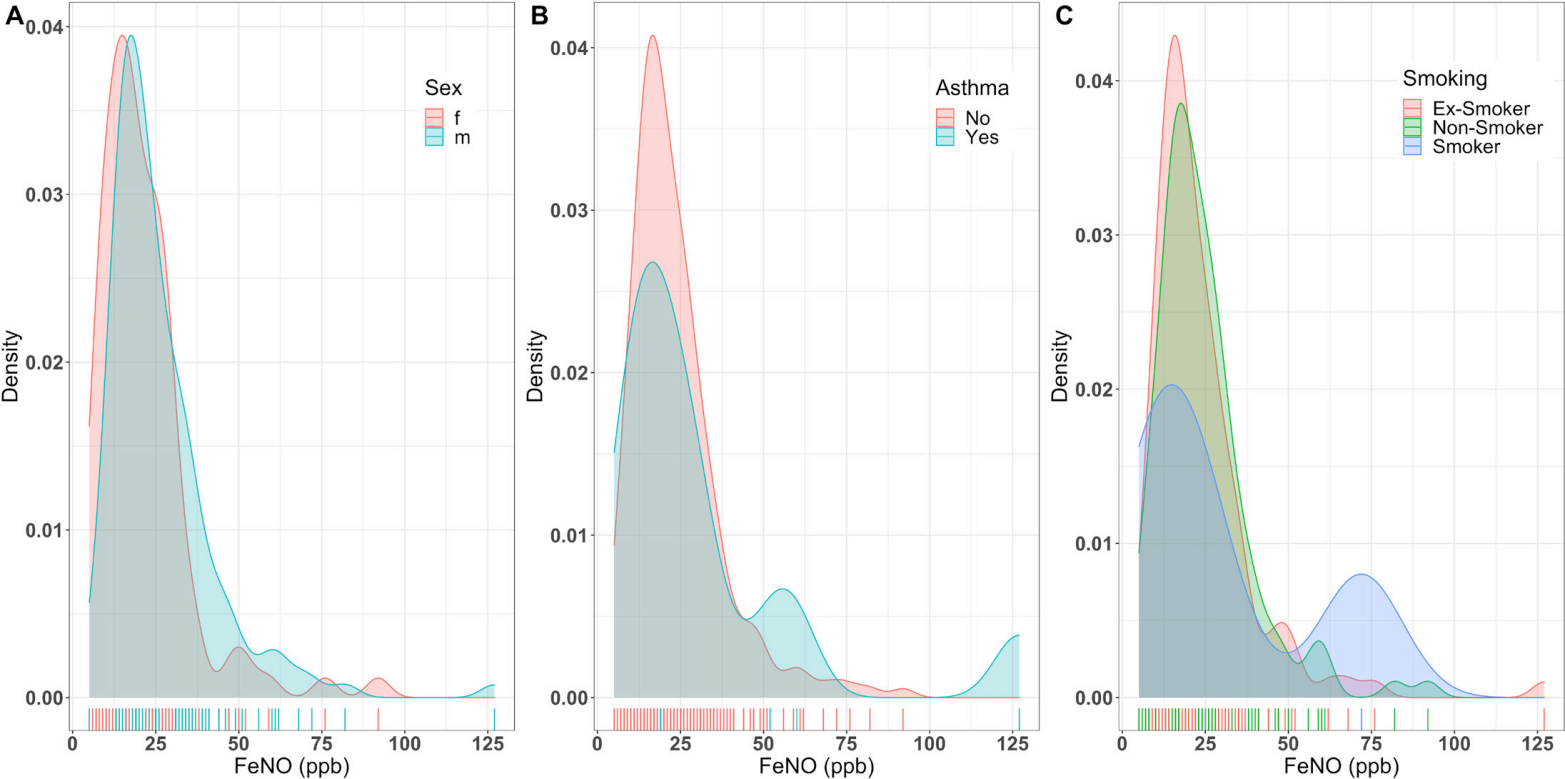
FeNO distribution according to clinical characteristics: A) distribution of FeNO stratified by sex; B) distribution of FeNO stratified by presence of asthma; and C) distribution of FeNO stratified by smoking status. f = female. FeNO = fractional exhaled nitric oxide. m = male. ppb = parts per billion.

**Figure 2.**
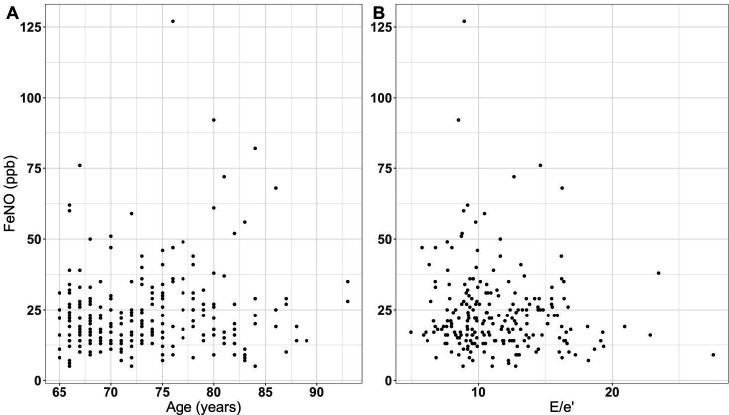
FeNO according to **A**) age, and **B**) E/e’ ratio. FeNO = fractional exhaled nitric oxide. ppb = parts per billion.

In those participants with a successful FeNO measurement, the mean FeNO was 24.2±15.6 ppb (median 20 ppb, interquartile range [IQR] 14–29). Selected variables previously observed to correlate with FeNO are illustrated in Figure 1, and the relationship between FeNO and age shown in Figure 2A. In the OxVALVE subset, only sex had a statistically significant association with FeNO (*P* = 0.003), with male participants having higher levels compared with females (median 22 and 18 ppb, respectively) (Figure 1). There was no correlation with age (univariate linear regression β 0.28, 95% confidence interval [CI] = –0.05 to 0.61, *P* = 0.09), and no statistically significant differences in FeNO in participants with asthma (*P* = 0.78), COPD (*P* = 0.45), or diabetes mellitus (*P* = 0.20), those using inhaled steroids (*P* = 0.82), and no relation to smoking status (*P* = 0.56).

### FeNO and echocardiographic variables

Echocardiographic measurements are displayed in Table 1 and demonstrate a median E/e’ ratio of 10.7 (IQR 9.1–13.5), which lies within the intermediate range (8–14) of population levels.^10^ Relationships between E/e’ and FeNO are displayed in Figure 2B and show no association of E/e’ ratio with FeNO (univariate linear regression β –0.03, 95% CI = –0.06 to 0.01, *P* = 0.10). As a sensitivity analysis, when assessment was restricted to those with breathlessness (NYHA class II–III, *n* = 99), there remained no significant association between E/e’ and FeNO (univariate linear regression β –0.08, 95% CI = –0.17 to 0.0, *P* = 0.06).

Clinical and echocardiographic characteristics of the normal, intermediate, and high E/e’ strata are displayed in Table 2, with mean FeNO levels of 23.8±11.4, 24.1±16.1, and 22.5±13.7 ppb, respectively (*P* = 0.74). Those with high E/e’ were older than those with normal E/e’ (76.8±7.4 years versus. 72.0±5.5 years, *P*<0.001), and the proportion of females in the high E/e’ range was greater than in the normal E/e’ range (58.6% versus 35.9%, *P* = 0.05). There were no other statistically significantly different clinical and echocardiographic characteristics across the E/e’ strata.

**Table 2.** Clinical characteristics of the OxVALVE subset stratified according to E/e’ ratio

Characteristic	Normal E/e’ (<8)	Intermediate E/e’ (8–14)	High E/e’ (>14)	*P* value
Participants, *n* ^a^	39	173	58	—
Age, years, median (IQR)	71 (68–76)	72 (68–77)	76 (71–83)	<0.001^b^
Sex, *n* (%)				0.045^c^
Male	25 (64.1)	100 (57.8)	24 (41.4)	—
Female	14 (35.9)	73 (42.2)	34 (58.6)	—
NYHA class, *n* (%)				0.098^c^
I	25 (64.1)	121 (69.9)	29 (50.0)	—
II	12 (30.8)	47 (27.2)	26 (44.8)	—
III	2 (5.1)	5 (2.9)	3 (5.2)	—
Ejection fraction, %, median (IQR)	69 (65–72)	69 (66–73)	68 (65–74)	0.97^b^
FeNO, ppb, median (IQR)	19 (17–31)	21 (14–29)	19 (14–27)	0.74^b^

^a^Seven (2.5%) participants did not have E/e’ measured. ^b^Kruskal-Wallis test for independent association of continuous variable with E/e’ strata. ^c^χ^2^ test for independent association of categorical variables with E/e’ strata. FeNO = fractional exhaled nitric oxide. IQR = interquartile range. NYHA = New York Heart Association. ppb = parts per billion.

## Discussion

### Summary

In this population cohort study of primarily asymptomatic participants, it has been demonstrated that there was no significant association between the echocardiographically derived E/e’ ratio (a reference standard for LAP) and FeNO. It is concluded that FeNO does not predict the presence or absence of HF in the primary care setting.

### Strengths and limitations

To the authors’ knowledge, this is the largest study to assess the presence or absence of an association between FeNO and E/e’. A major strength of the study is that investigation was undertaken in primary care where such a test would prove most useful. In addition, the study was conducted prospectively in consecutive participants, and OxVALVE participants are representative of the wider primary care population.^11^ Furthermore, participants were aged ≥65 years, the cohort most frequently affected by HF.

The cross-sectional design of this study meant important day-to-day intra-individual variation in expired nitric oxide was not assessed.^12^ In addition, potential confounders, such as caffeine and alcohol consumption, were not recorded, limiting the ability to adjust for these variables.^13,14^

FeNO measurement was unsuccessful in 18.1% of participants tested. Reasons for unsuccessful measurement were mainly related to inability of the patient to follow instructions or maintain constant expiration of approximately 50 ml/s. Consistent with a previous study,^15^ the present study demonstrated that FeNO measurement was not feasible in all individuals, which limits its use as a diagnostic test.

Participants from the OxVALVE population cohort were recruited from their local general practice (rather than hospital inpatients or outpatients) and were consequently relatively healthy. However, this is the population in whom a simple screen for LAP would have greatest value. There were low numbers of current smokers (2.9%), participants with asthma (7.2%), COPD (5.8%), or cardiovascular risk factors such as diabetes (13.7%), which may have reduced study power. Furthermore, there is a significant difference in the severity of population-level asthma, which may be controlled or seasonal, and that of patients with asthma presenting to secondary care. Previous associations of FeNO with conditions such as asthma may not be relevant in less severe disease.^16–18^

### Comparison with existing literature

The mean FeNO in this cohort was 24.2 ppb, which is slightly higher than that observed in previous population studies using a flow rate of 50 ml/s. Population studies in Japan,^19^ Germany,^20^ the US,^21^ and New Zealand^22^ all observed lower mean FeNO. However, these studies were in younger cohorts than the OxVALVE subset. A previously reported association between older age and increased FeNO may explain the differences in mean FeNO between studies.^20^ The present study aligns with previous population studies that demonstrated higher mean FeNO levels in males compared with females.^19,21–23^

Previous research has suggested an association between various clinical conditions and FeNO, including asthma,^16–18^ COPD,^24,25^ and diabetes.^26^ Current and previous smoking habit,^23,25,27^ inhaled steroid use,^28–30^ and low and high body mass index (BMI)^31^ have also been associated with low FeNO. However, no such associations were observed in the OxVALVE subset.

Previous smaller studies have suggested a relationship between FeNO and elevated LAP. A study of 34 chronic symptomatic patients with HF found increased FeNO levels post-exercise, although no association with echocardiographic measures was observed at baseline.^6^ Conversely, a study comparing compensated (*n* = 30) and decompensated (*n* = 7) patients with HF to healthy controls (*n* = 90) observed increased FeNO in patients with HF.^32^ In addition, a study comparing participants with rheumatic heart disease (*n* = 74) and healthy controls (*n* = 27) found significantly increased FeNO in patients with pulmonary hypertension,^7^ suggesting a relationship between pulmonary congestion and FeNO (since pulmonary hypertension is almost universally because of high LAP in rheumatic heart disease). However, these studies were undertaken in selected younger cohorts with HF and rheumatic heart disease. OxVALVE is a population-based study in participants aged ≥65 years and the lack of any association between FeNO and E/e’ ratio may be more relevant in an unselected older population.

### Implications for research and practice

This study contributes to current research that examines the relationship between FeNO and LAP, with particular focus on the older population, a group that bears the greatest burden of morbidity and mortality related to HF. Despite evidence suggesting elevated FeNO may be a marker of LAP, the present study provides evidence that there is no significant relationship between the two. As such, FeNO would not be a clinically appropriate test for elevated LAP in HF within this patient cohort. There remains an unmet need for improved assessment of potential patients with HF in the primary care setting. In patients with previously diagnosed HF, the clinical decision as to whether adjustment of current management approach would improve symptoms is often difficult to assess, with complex interactions between cardiac and non-cardiac contributors to symptoms.^33^ Beyond the assessment of symptoms, there has also been a move towards more objective methods to guide management.^34^ Natriuretic peptides may help in this scenario, but require additional patient contact for blood testing and adjustment of treatment. Further research should examine whether bedside testing (using point-of-care ultrasound to assess the lungs or inferior vena cava, for example) would help to refine clinical management. In conclusion, FeNO is not an accurate predictor of elevated E/e’ in predominantly asymptomatic primary care patients.

## Data

Not publicly available.
